# Supplementary material for: Picroscope: low-cost system for simultaneous longitudinal biological imaging
Source: Commun Biol. 2021 Nov 4;4:1261. doi: 10.1038/s42003-021-02779-7 (PMC8569150; doi:10.1038/s42003-021-02779-7)
Supplement: Supplementary file 1 — Supplementary Material [file 42003_2021_2779_MOESM1_ESM.pdf]

Communications Biology
Supplementary Information

Picroscope offers Low-Cost System for Simultaneous Longitudinal Biological Imaging

Victoria T. Ly, Pierre V. Baudin, Pattawong Pansodtee, Erik A. Jung, Kateryna Voitiuk, Yohei M. Rosen, Helen Rankin Willsey, Gary L. Mantalas, Spencer T. Seiler, John A. Selberg, Sergio A. Cordero, Jayden M. Ross, Marco Rolandi, Alex A. Pollen, Tomasz J. Nowakowski, David Haussler, Mohammed A. Mostajo-Radji, Sofie R. Salama, and Mircea Teodorescu

Supplementary Video 1: Video Imaging of a Developing Zebrafish
Supplementary Figure 1: Comparison of illumination options in different sample types.
Supplementary Figure 2: Imaging a resolution test target (USAF 1951) and Field of View (FOV).
Supplementary Figure 3: Thermal Images of the Picroscope during operation.
Supplementary Note 1: Microscope Assembly Guide

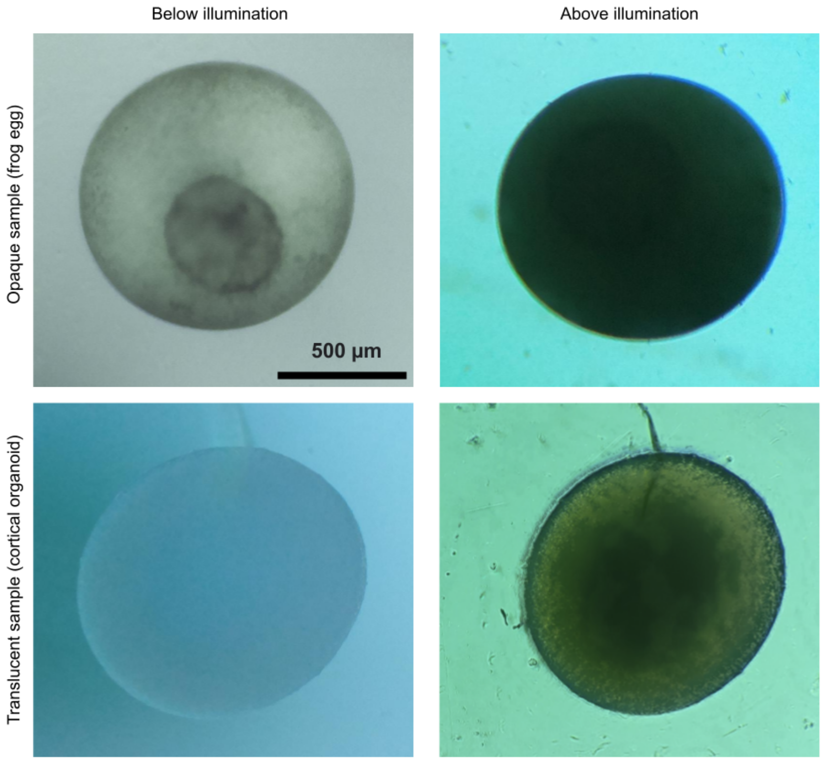

Supplementary Figure 1. Comparison of illumination options in different sample types. Comparison between above and below illumination for imaging opaque samples (frog egg) and translucent samples (cortical organoid).

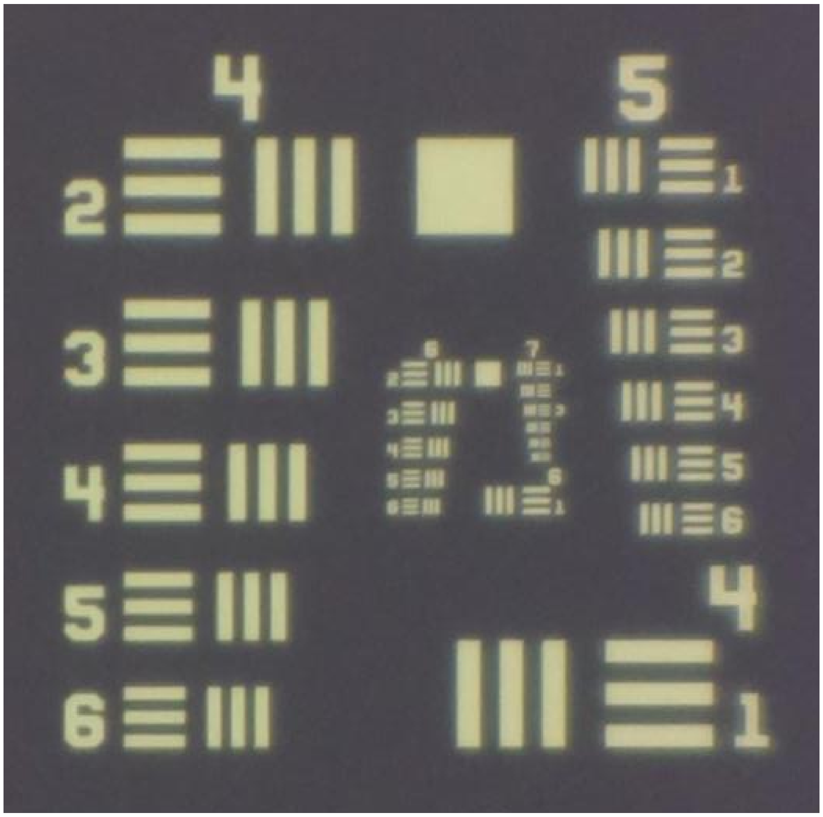

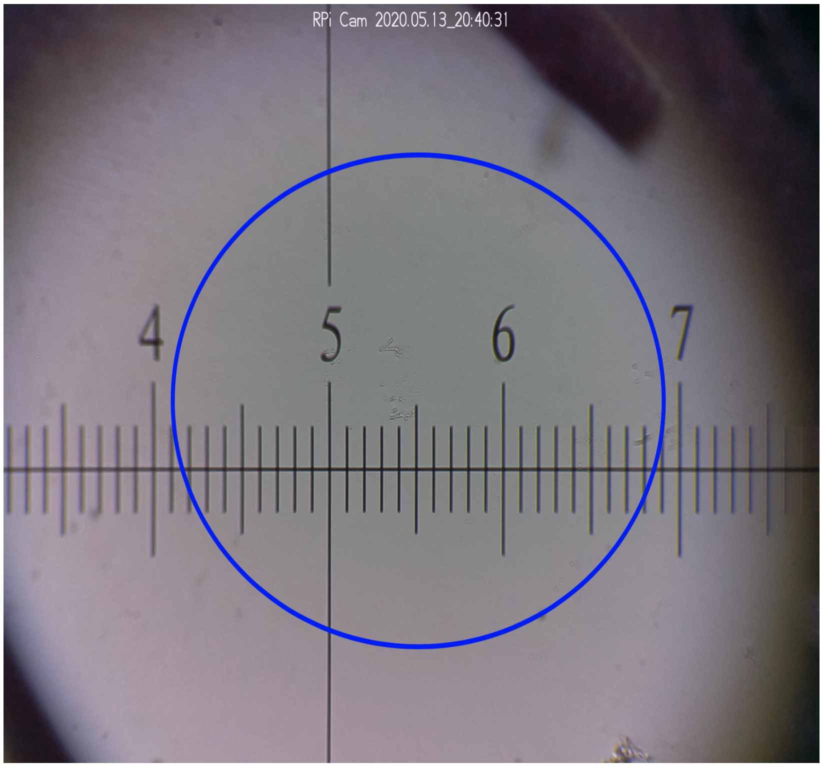

Supplementary Figure 2. Imaging a resolution test target (USAF 1951) and Field of View (FOV). We concluded the resolution to be group 7 element 1, 128.00 lp/mm, roughly equally 7μm. The image below shows the field of view as well as the portion of the image (in the center) that has been considered for analysis here.

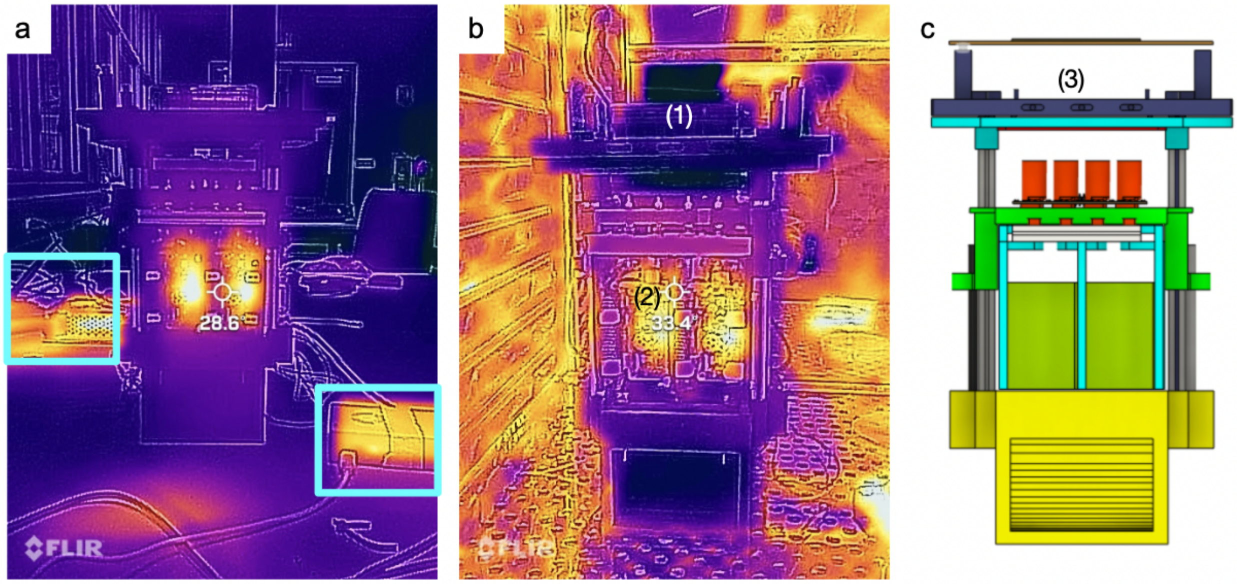

Supplementary Figure 3. Thermal Images of the Picroscope during operation. a. The Picroscope on a lab bench. The two blue boxes indicate components that will not be placed in the incubator. b. The Picroscope during operation inside of an incubator. The temperature of the 24 well plate (1) is not affected by the heatsource (2) c. CAD rendering of the Picroscope (3) indicates the cell plate holder

Supplementary Note 1: Picroscope Assembly Guide

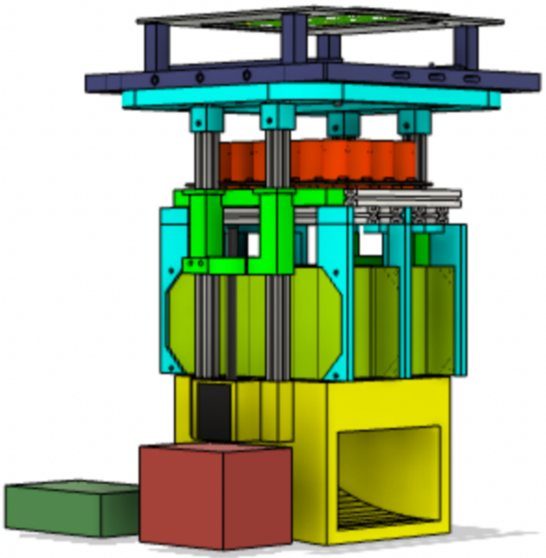

Table of Contents

List of components: 5

- Structural components: 5
  - General notes: 5
  - List of 3D printed and CNC machined components 5
  - MakerBeam Components & Screws 5
- Electronic Components: 5
- Assembly Guide for Electronic Custom Power Distribution Boards 6
- Optics: 6
- Coating & Glue 6

Assembly Guide 7

- General notes: 7
- The Picroscope assembly Base 7
- The Elevator stage 8
- Camera Unit 9
- XY Stage and Overhead lighting 11
- Wiring Guide 12
- PCB Assembly guide 12
  - PCB reference 13
  - Soldering Guide 16
  - Pi hub assembly 21
  - Wire assembly 26

List of components:

Structural components:

General notes:

- All files can be found on the following Github Repository https://github.com/braingeneers/picroscope-supplement/.
- All of the 3D printed components were fabricated using a PRUSA MK3S Printer with 100% infill using the PrusaSlicer.

List of 3D printed and CNC machined components

- (1) Base (3D print)
- (2) Right PCB Frame holder (3D print)
- (2) Left PCB Frame Holder (3D print)
- (2) Middle PCB Frame Holders (3D print)
- (1) Elevator (3D print)
- (4) Camera Rows* (printed at 0.10mm quality or higher) (3D print)
- (24) Camera Shunts (3D print)
- (1) XY Stage Base (3D print)
- (1) XY Stage + cell plate holder (3D print)
- (2) Overhead light holders (3D print)
- (1) Under light raiser (3D print)
- (1) Acrylic diffuser (CNC)
- (1) Acrylic over head light holder (CNC)

MakerBeam Components & Screws

All of the screws are MakerBeam M3 unless otherwise stated.

- (4) 200mm Bars
- (4) 150mm Bars
- (2) 100mm Bars
- T nut screws
- M2.5 Heat insets

Electronic Components:

- (1) Raspberry Pi 4 + USB-C Charger (must be the official Raspberry Pi version)
- (24) Raspberry Pi Zero W
- (25) 32GB microSD cards

- (1) Arduino Uno
- (1) V2 Arduino Motor Shield
- (24) Spy Cameras for Raspberry Pi (5 Mp)
- (2) Limit switches
- (3) RPI0W Custom Power Boards
- (1) Custom Under Illumination Board (pat insert led type)
- (1) Custom Over illumination Board (pat insert led type)
- (1) Router
- (1) USB 2.0 Printer Type Cable - A-Male to B-Male
- (1) Ethernet cable
- (2) Nema 11 External 34mm Stack 0.75A Lead 0.635mm/0.025" Length 100mm
- (1) Fan AXIAL 80X25MM 05VDC
- (1) Custom Relay Board
- (1) 5W Power Supply

Assembly Guide for Electronic Custom Power Distribution Boards

- The system uses 3 identical printed circuit boards, which are configured differently, as explained below.

Optics:

- (24) Spy Cameras for Raspberry Pi (5 Mp)
- (24) Arducam 1/2.5" M12 Mount 16mm Focal Length Camera Lens M2516ZH01

Coating & Glue

- LOCTITE 4851
- Super Corona Dope Varnish

Assembly Guide

General notes:

- Fabricate (3D print or CNC) the components listed above.
- Solder (3) 2x3 male headers on each Raspberry Pi Zero W (24) as shown in Figure SN1.

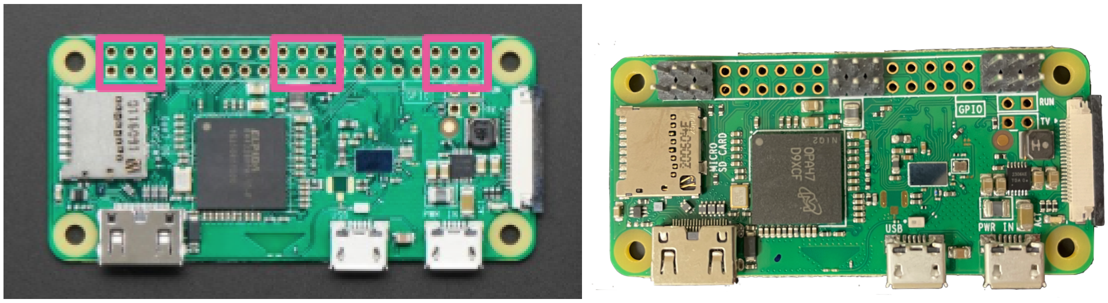

Figure SN1: Raspberry Pi Zero W with and without pin headers.

- Using Balena Etcher, download the clone of the Raspberry Pi Zero W software we provided on the Github drive and copy it onto micro SD cards (x24).
- Download the clone for the Raspberry Pi 4 (hub Pi) (x1). Once the microSD card has the clone, you must individually go in and update the hostname of each pi as well as the hubname (ie A11, A12 ...A16, A21 - A26, A 31 - A36, A41 - A46 [in this example “A” corresponds to the hub pi, the first number corresponds to the row, and the second corresponds to the column position of the specific Raspberry Pi]).
- Coat each Raspberry Pi (front and back) with the Super Corona Dope Varnish (avoid the camera connector, you will need to access this part later)
- Solder the female headers into the custom power PCBs. (if they are not already soldered)

  NOTE: We recommend labeling the Raspberry Pi computers as well and keeping them in order to make the rest of this assembly easier!

The Picroscope assembly Base

- Insert the fan in the 3D printed base and secure it using M3 Screws.
- Attach both motors on each side of the base using M2.5 Screws (~4mm length).
- Insert the four vertical MakerBeam extruded profiles (section 10x10mm, length 200m) in the 3D printed base and attach with four maker beam screws. See Figure SN3.

The Elevator stage

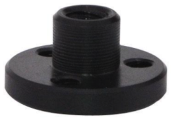

- Remove the black threaded flanges shown in Figure SN2 from each of the two motors and insert them into the elevator piece.

- Attach the flanges using M3 screws and M3 locking nuts.
- Attach the limit switch (see figure SN3)

Figure SN2:
Threaded flange

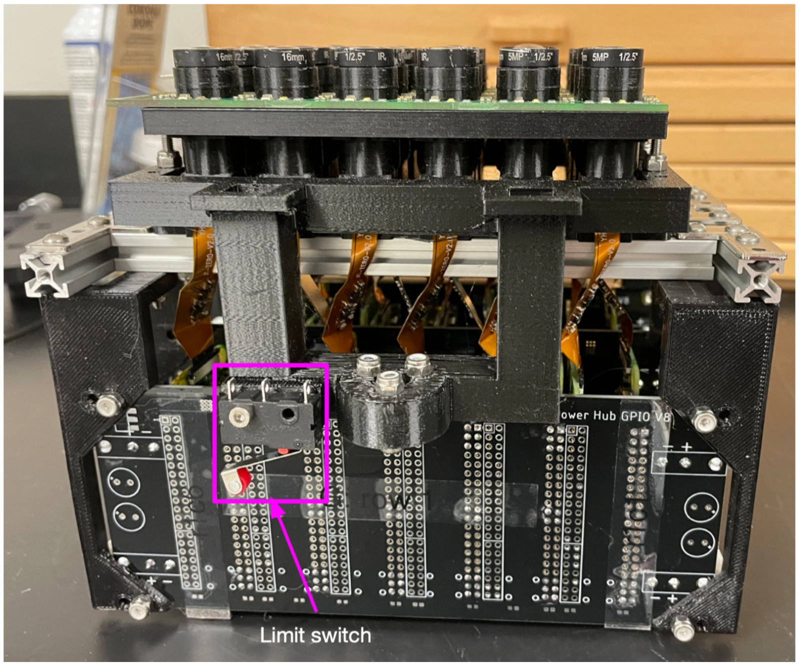

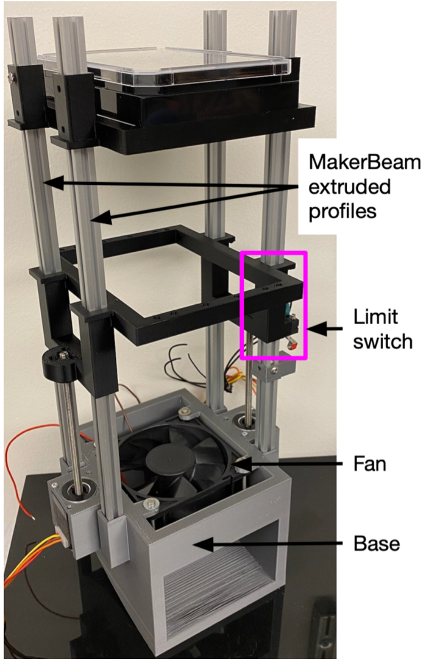

Figure SN3: (left) The assembled elevator stage (right) Fan and base assembly

Camera Unit

Overview: Figure SN4 shows the assembled of the camera units

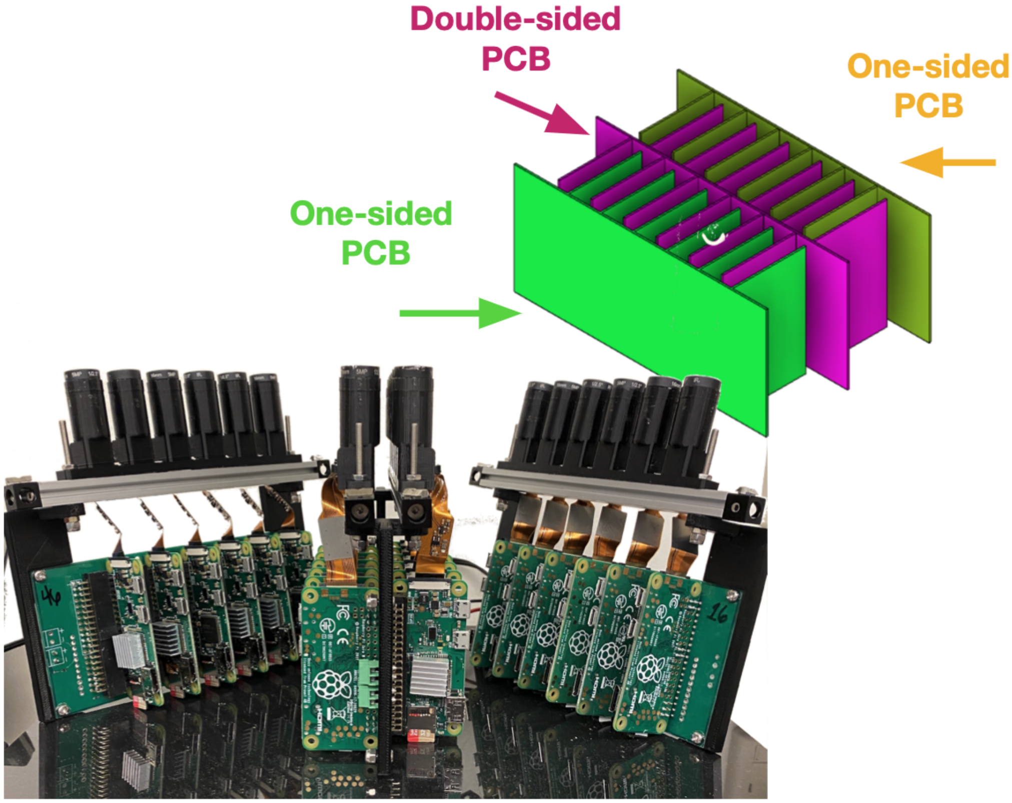

Figure SN4: Full Camera, Raspberry Pi Zero W, power board assembly

- The Raspberry Pi Zero W computers with the header pins soldered in should be inserted into the custom power boards. There are in total 3 power boards. One double sided board, and 2 single sided boards.
- Attach the PCB boards to the lower side of the Maker Beam profiles using the 3D printed holders (see Figures SN4-6).
- Attach the camera camera rows to the upper side of the MakerBeam profiles (see Figures SN4-6).
- Screw in the lenses (Arducam 1/2.5" M12 Mount 16mm). One for each barrel (see Figures SN4-6).

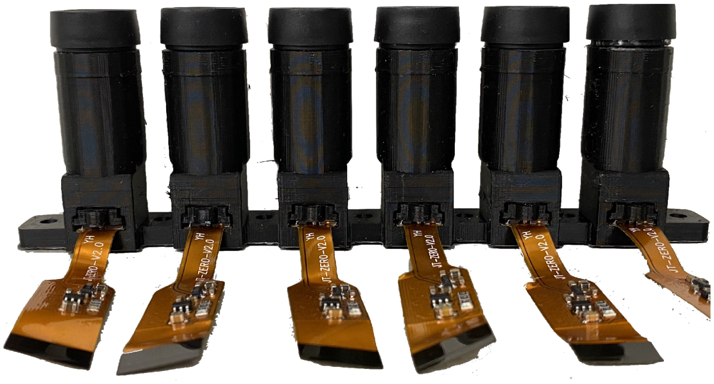

Figure SN5: One row of camera holders (the camera connexion cables are visible)

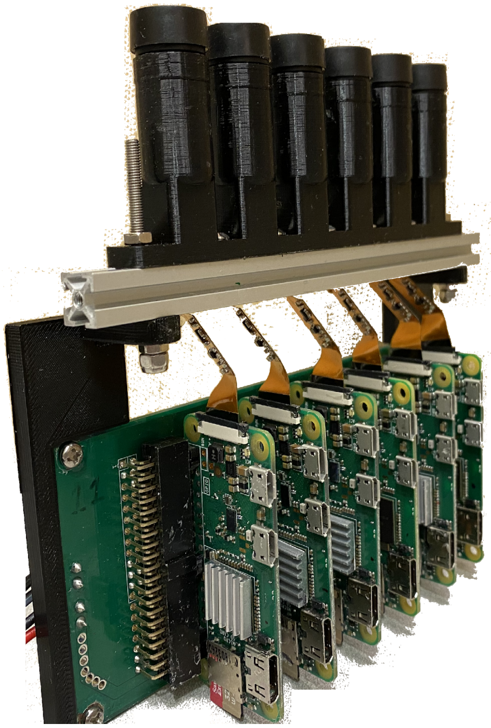

Figure SN6: One completed power board and camera holders

- Remove the lenses from all of the 24 Spy Cameras for Raspberry Pi (e.g., using tweezers)
- Insert the camera units into the camera orifices at the bottom of the camera barrels as far back as possible and close secure each one with a small 3D printed shunt piece.
- Secure the camera rows to the beams using two M3 screws
- Attach each spy camera cable to the corresponding Raspberry Pi Zero W and secure it with LOCKTITE 4851.
- Coat each board with the Super Corona Dope Varnish.
- Assemble the boards on the elevator piece using the interlocking configuration as shown in Figure SN7.
- Secure the three MakerBeam profiles (supporting the 4 rows of cameras) using two additional perpendicular.
- The final elevator piece is shown in Figure SN3.

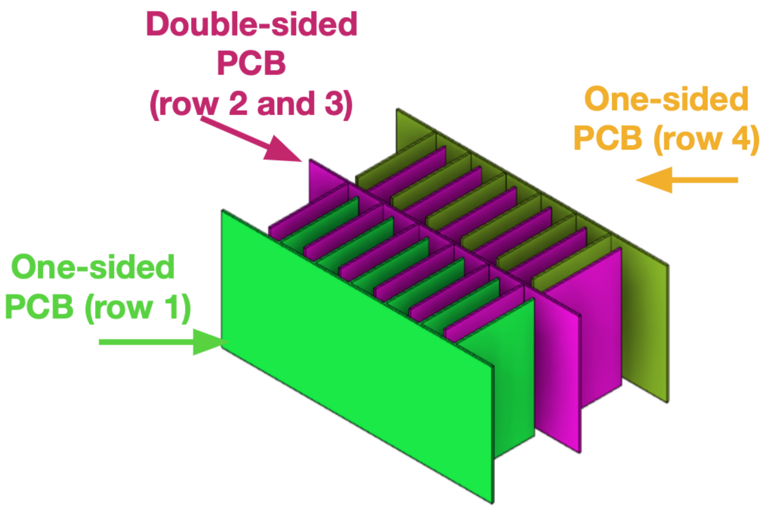

Figure SN7: Interlocking design of how the Raspberry Pi Zero W fit together

XY Stage and Overhead lighting

- Glue the overhead light holders onto the XY stage using LOCTITE 4851.
- Ziptie the two components of the XY Stage.
- Attach the overhead PCB to the acrylic overhead light holder.
- Using plastic m3 standoffs, attach the acrylic overhead light holder to the 3D printed overhead light holder. This can be adjusted by adding more or less plastic standoff components.

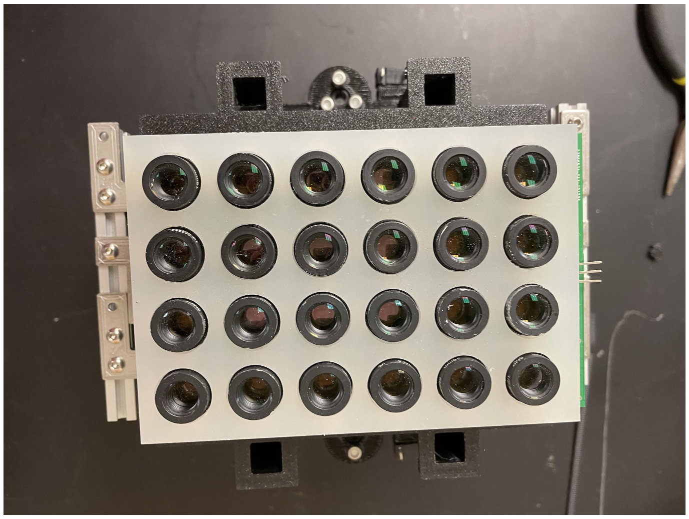

Figure SN8: Above view of camera units one assembly is complete

Wiring Guide

- Plug in the wires for the motors into the Arduino Motor shield. From left to right should go yellow, orange, brown, red.
- The Custom Relay board, sits on top of the Arduino Motor shield (which sits on top of the Arduino Uno)
- Plug in according to the labeling on the Custom Relay Board

PCB Assembly guide

Download PCB design files: https://github.com/braingeneers/picroscope-supplement/.

1. For the relay board and below light board use JLCPCB. For the above light board use PCBWAY.
2. Follow the JLCPCB assembly order: https://youtu.be/MICBFN2mD6Q?t=717
3. Order from PCBWay: https://www.pcbway.com/helpcenter/Findproducts/How_do_I_place_an_order_.html
    a. click "quick-order" on the top right and upload the files from **Gen5 > Power_distribution > pinheader_v8** . The parameters will be automatically be filled in. Changes:
        i. solder mask color = black
        ii. Quantity = 20
        iii. Can’t be ordered populated and you need to do the credit card

PCB reference

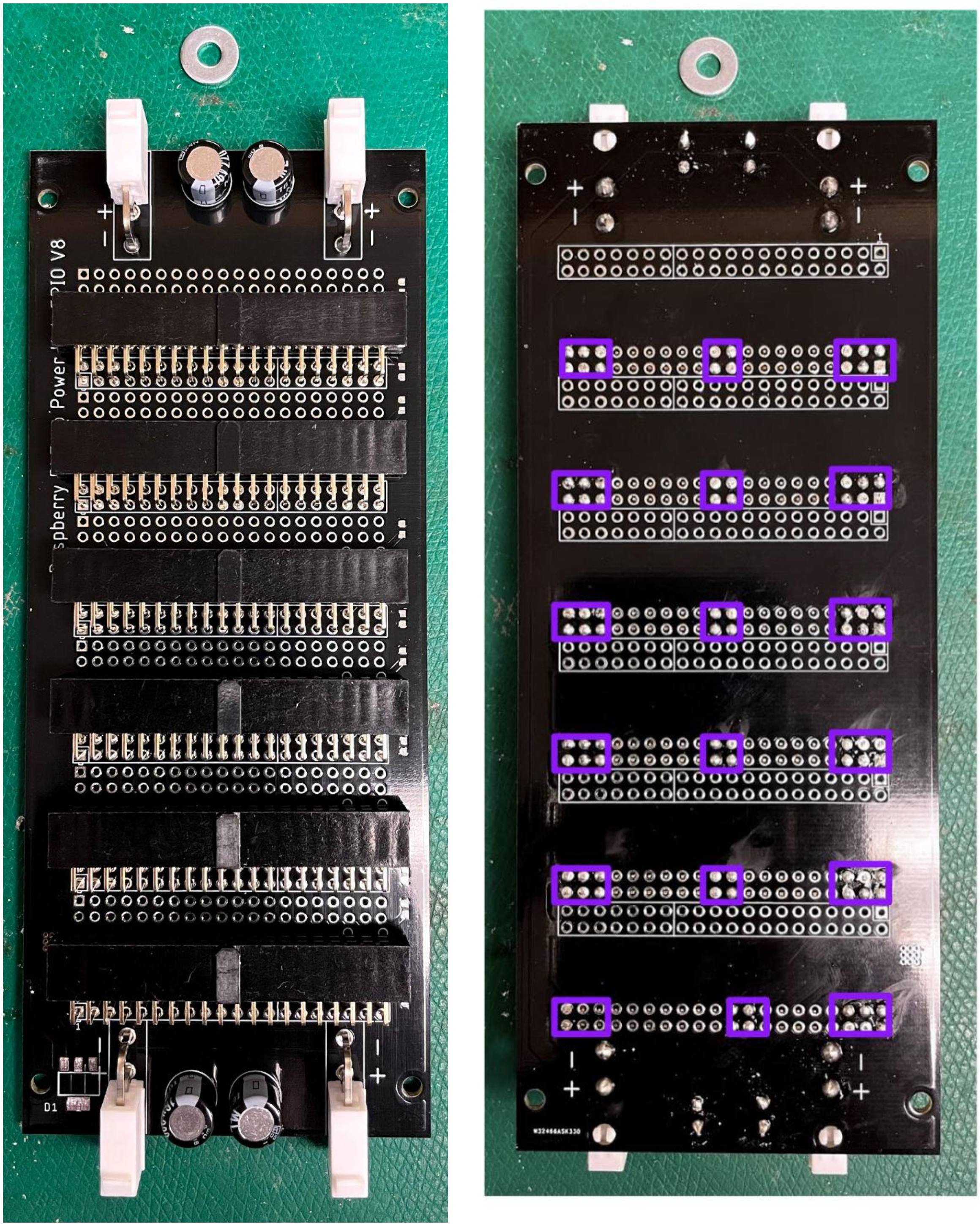

Figure SN9: Left PCB : Top and Bottom (Purple: required soldering locations)

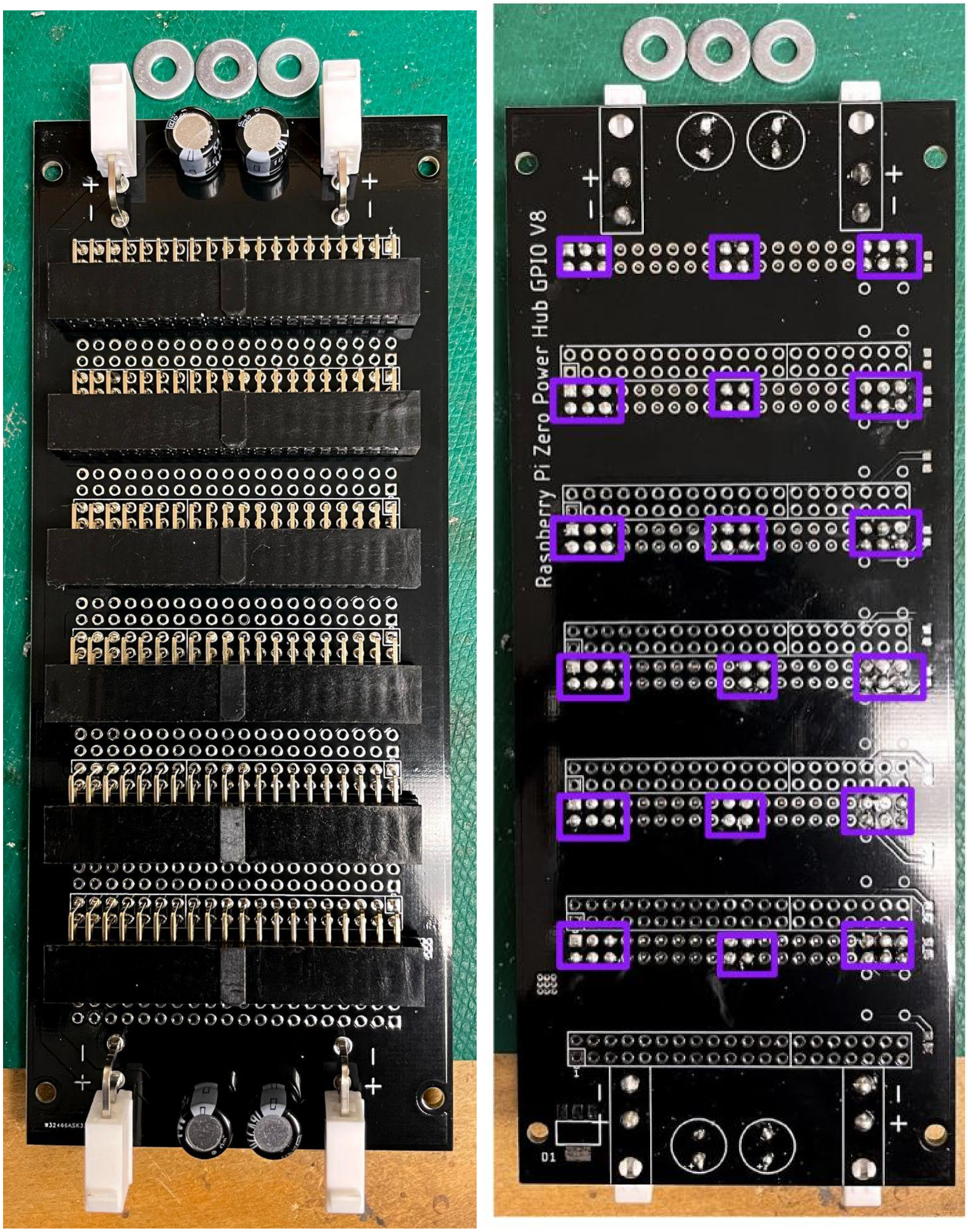

Figure SN10: Right column PCB Top(left picture) and Bottom (right picture) where the purple boxes show the required soldering locations.

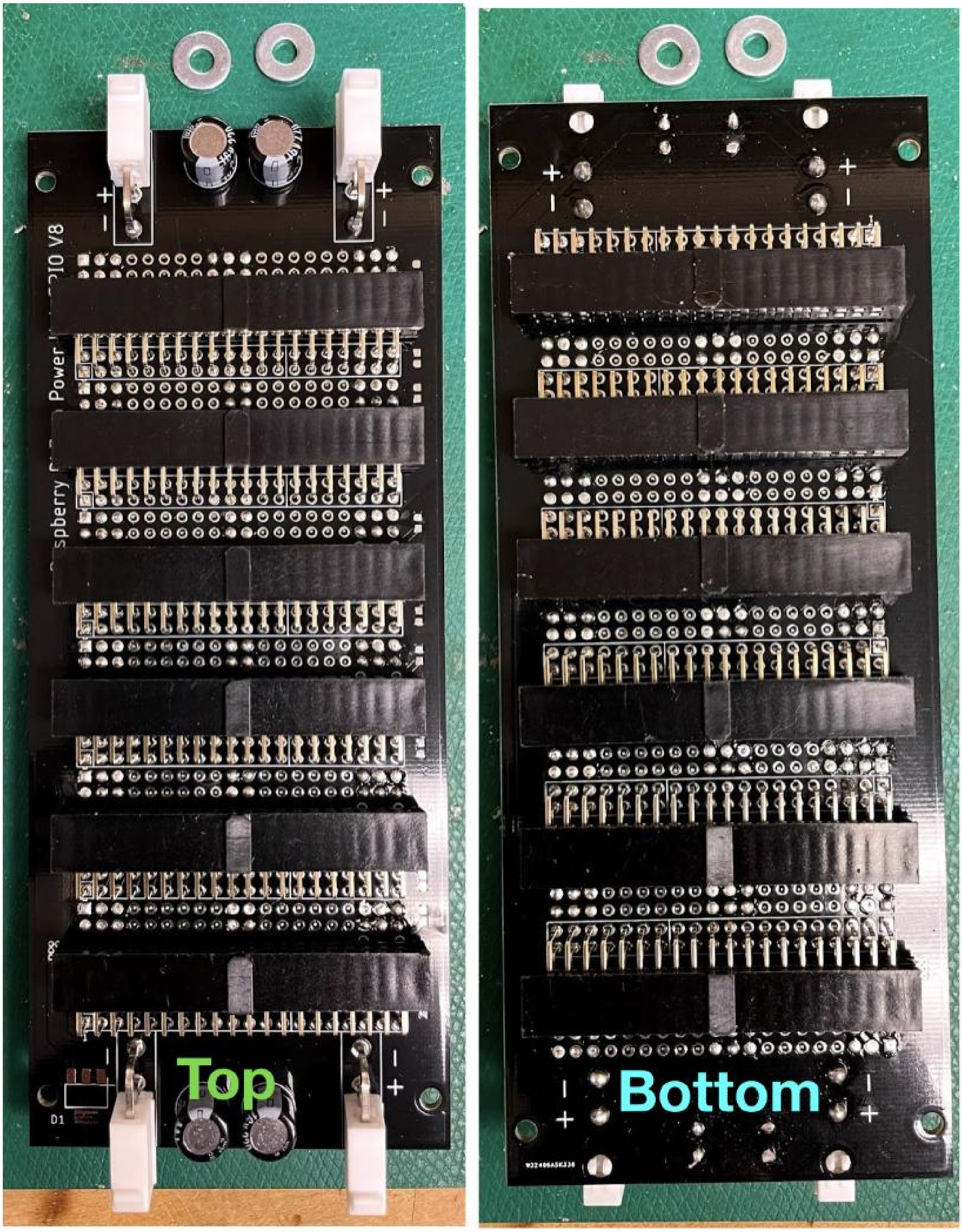

Figure SN11: Middle PCB Top(left picture) and Bottom (right picture) show the finished product.

Soldering Guide

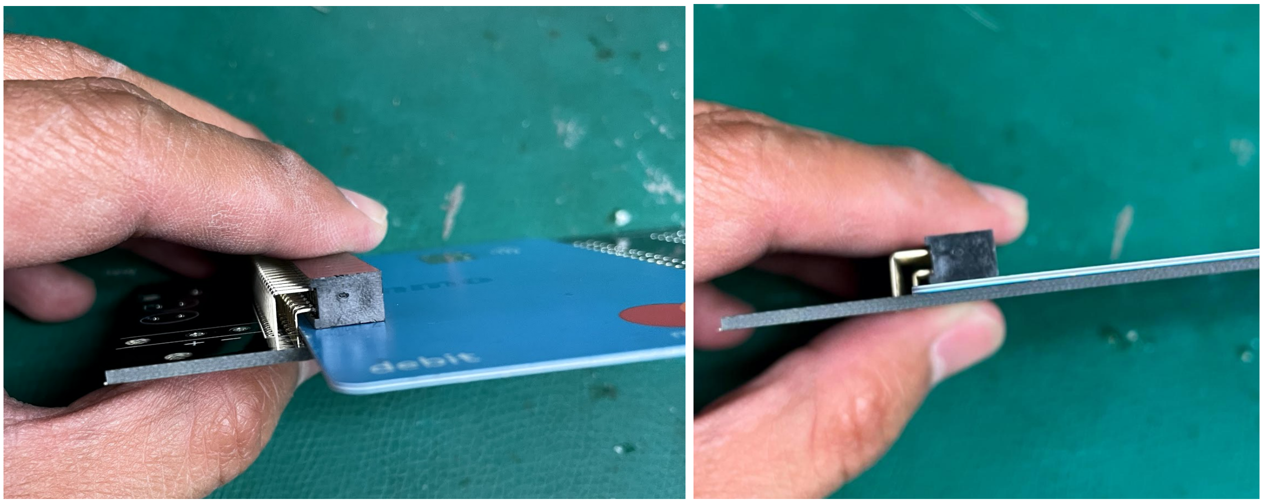

Figure SN12: Step #1 Place SFH11-PBPC-D20-RA-BK connector into a slot (left) and use a credit card to raise the connector before soldering (right).

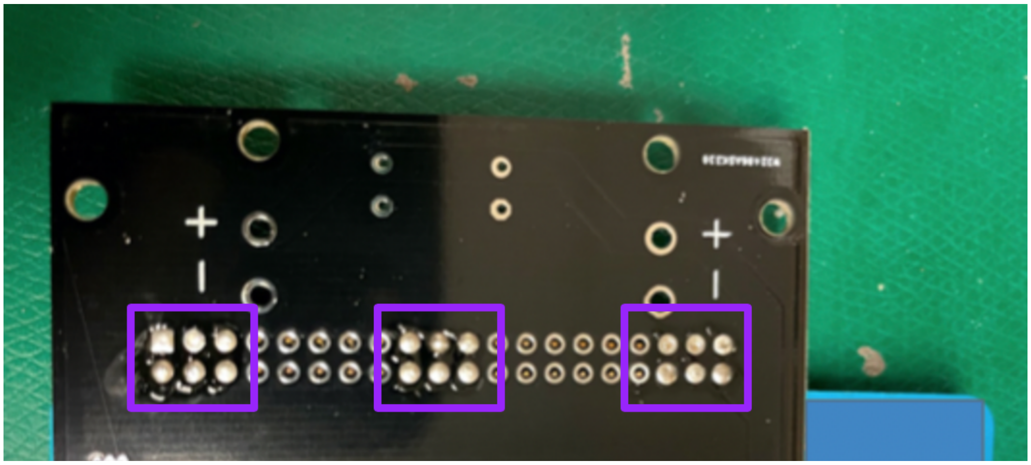

Figure SN13: Step #2 Soldering 6 pins (like the purple boxes) for the left, middle and right

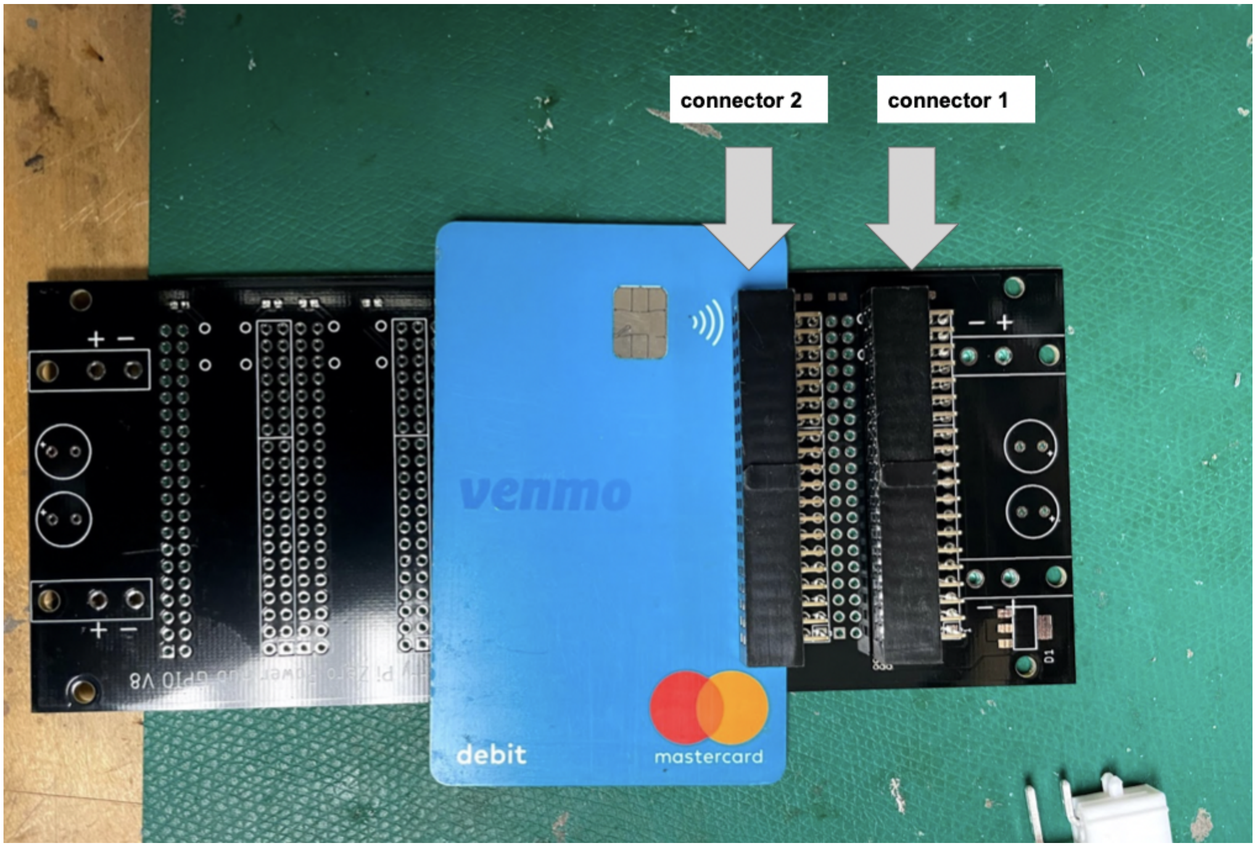

Figure SN14: <u>Step #3</u> Soldering the rest of the connectors.

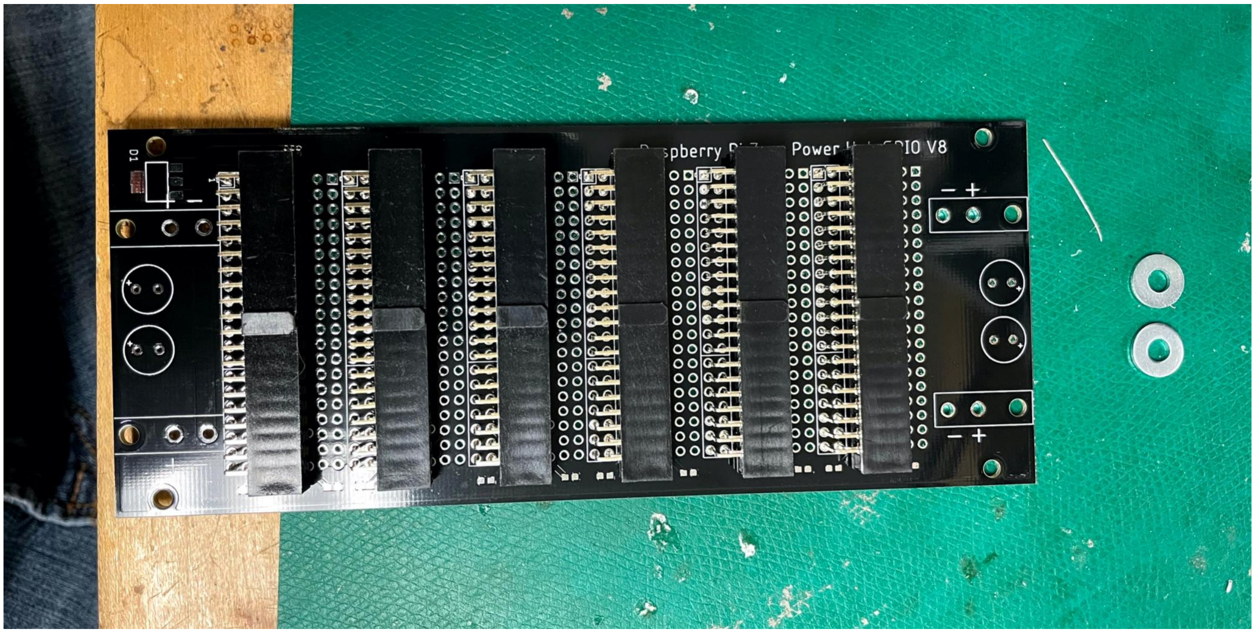

Figure SN15: <u>Step #4</u> One side is complete!

Step #5-7 are ONLY for the middle PCB

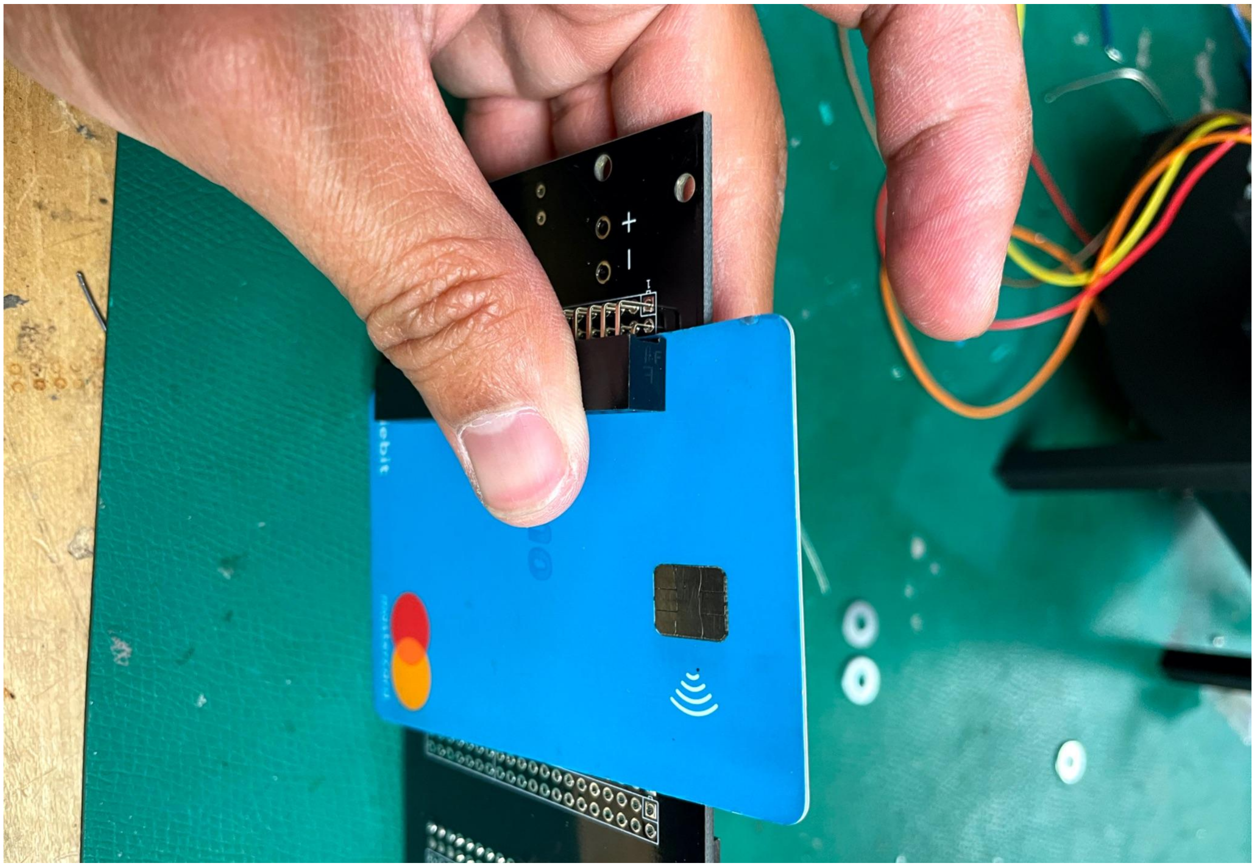

Figure SN16: Step #5 Insert the connector on the other side, and use a credit card to raise the connector gap.

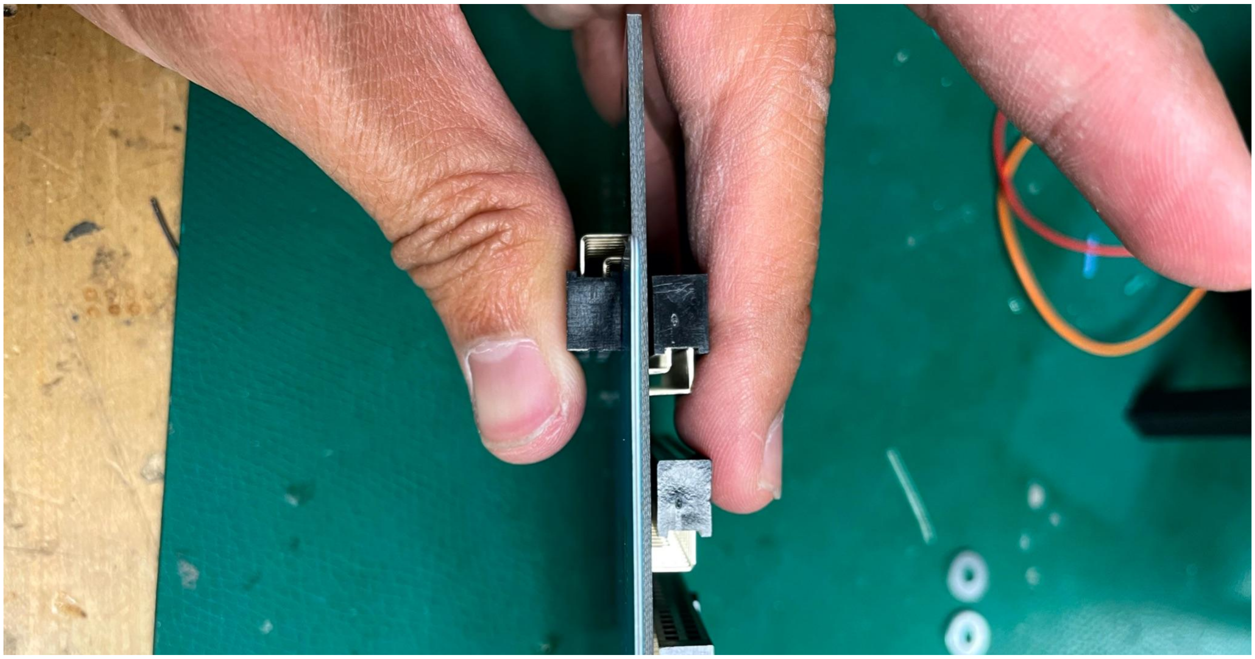

Figure SN17: Step #6 Avoid touching the soldering iron to the black plastic piece and make sure it is still lifted properly.

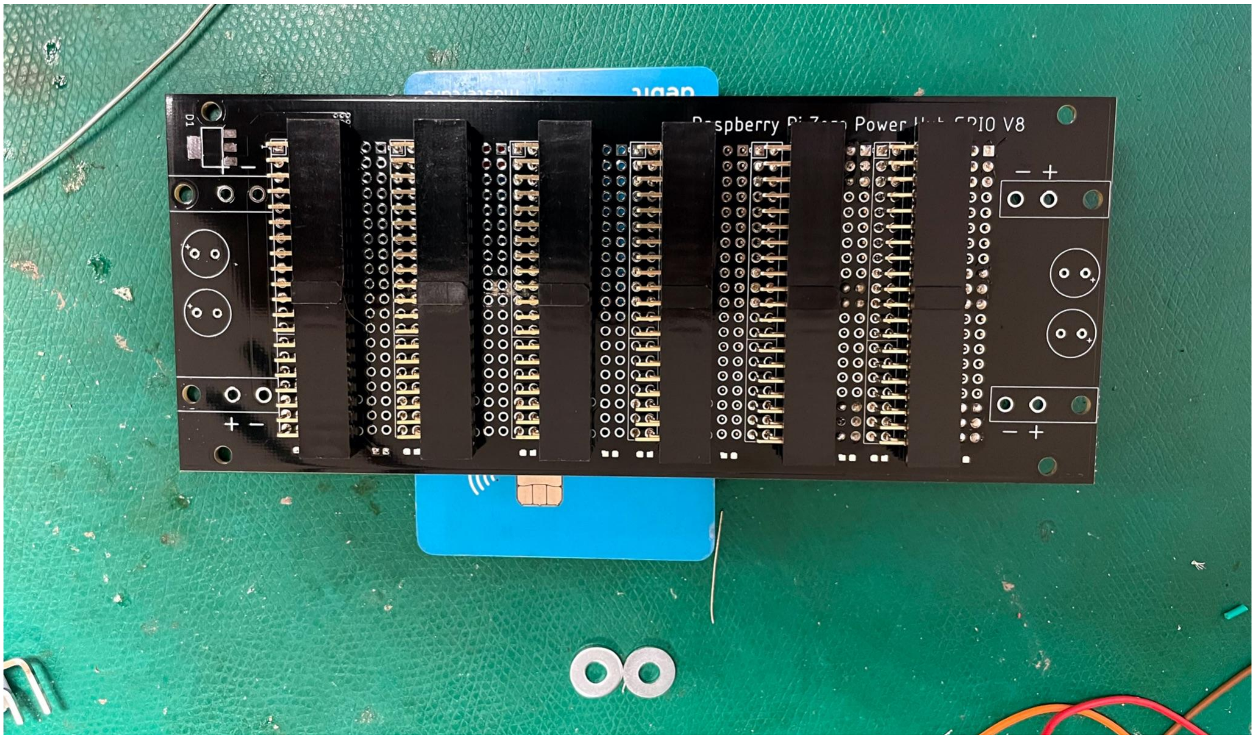

Figure SN18: Step #7 Continue until the PCB is fully populated.

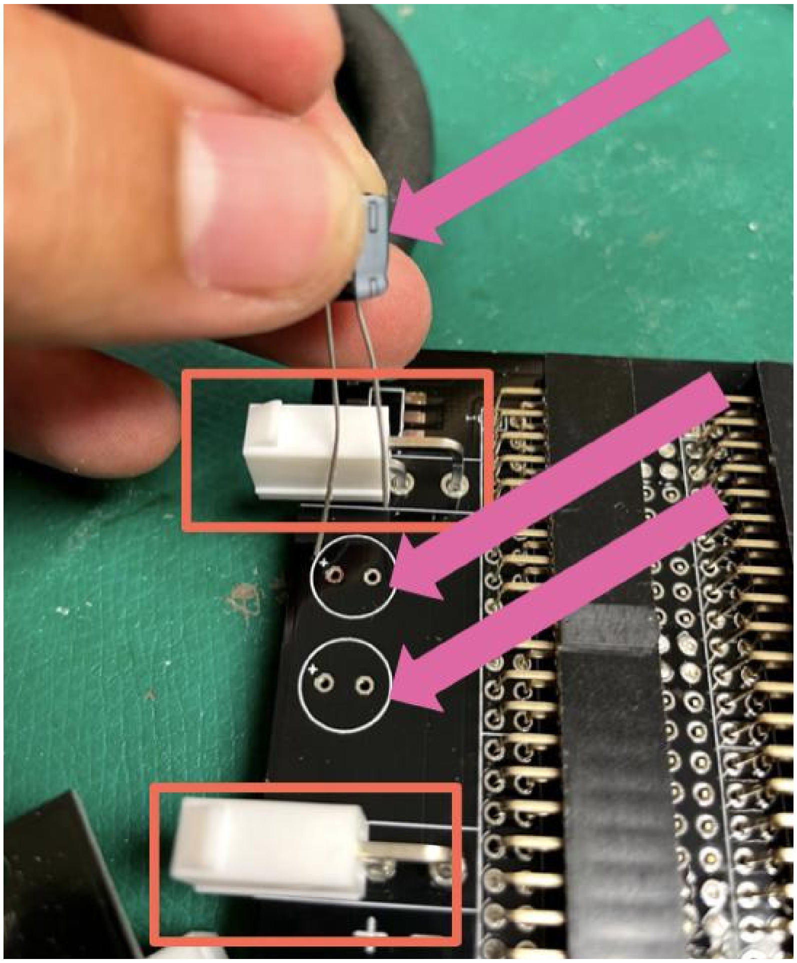

Figure SN19: Step #8 Solder the power connectors (part number: wm21363-nd, 0039301022) [red rectangles] and capacitors (Part number: 16ML220MEFCT78X7.5) [pink arrows]

Pi hub assembly

Please see the linked files on GitHub.

Step #1 Order gen5/pi_relay_hub/v3r1/ from JLCPCB pre-populated using the following filing option:
(a) Black PCB
(b) Use the Bill of Materials (BOM) in the following file: jlc_bom.csv
(c) Use the Part placement (PNP) in the following file: jlc_pnp.csv
(d) SMT Assembly needs to be toggled and tools assembled by JLCPCB

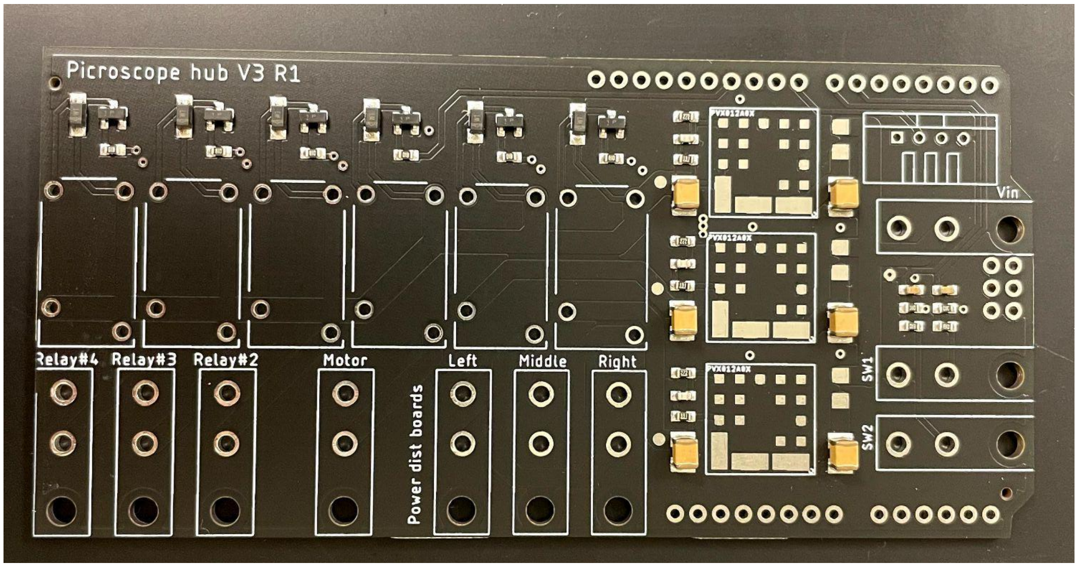

Figure SN20: This is a pre-populated PCB from JLCPCB.

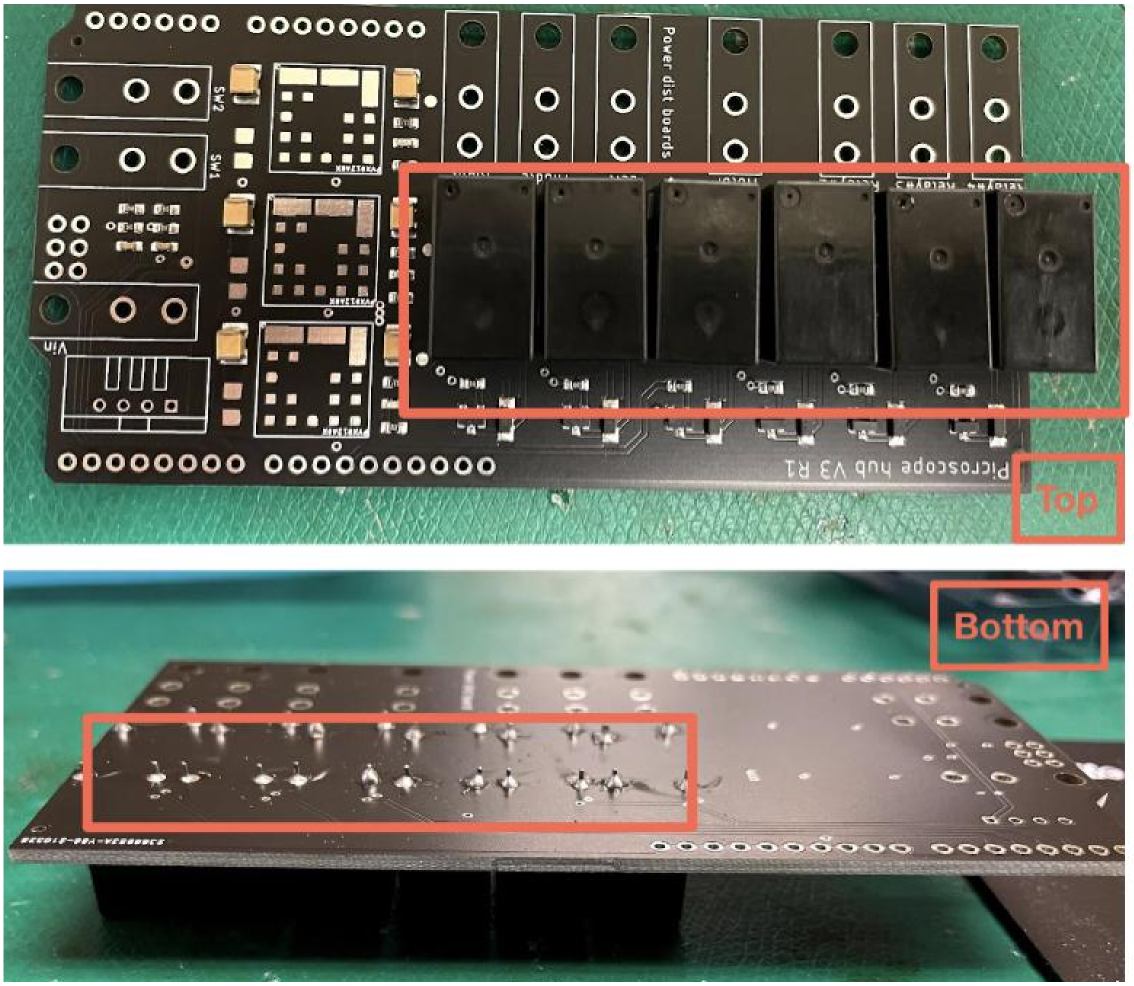

Figure SN21: Step #2 Solder 6 relays (Part number: OJT-SS-112HM, 0000)

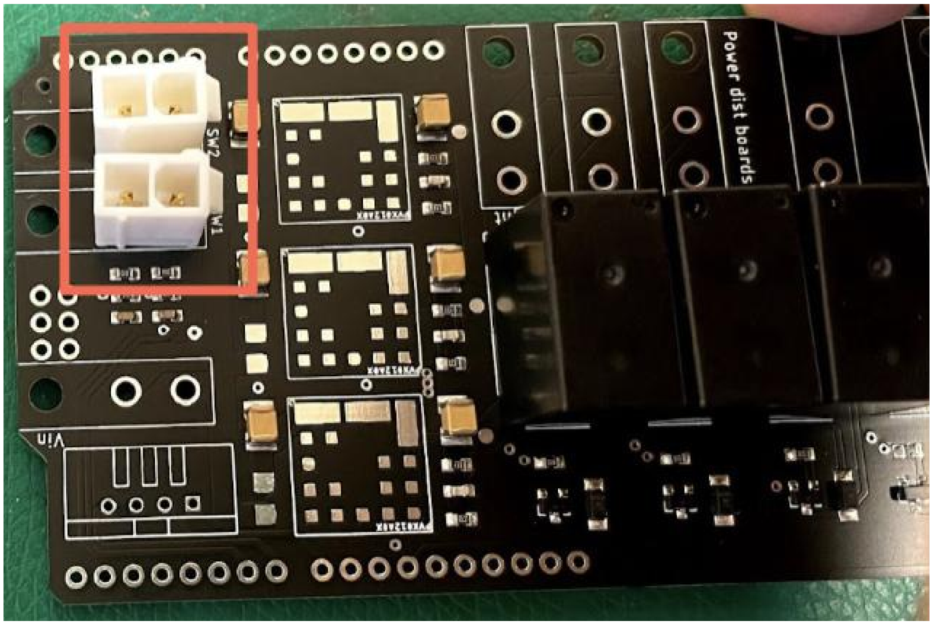

Figure SN22: Step #3 Solder 2 limit switch connectors (Part number: 0039296028) where the red box is located.

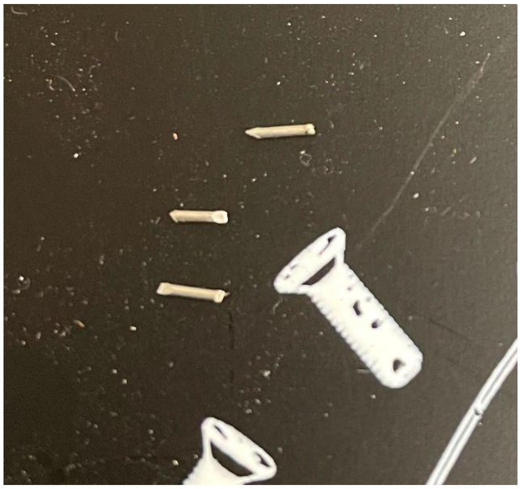

Figure SN23: Step #4 Cut three wires into 5 mm length each. We recommend using the legs of a resistor.

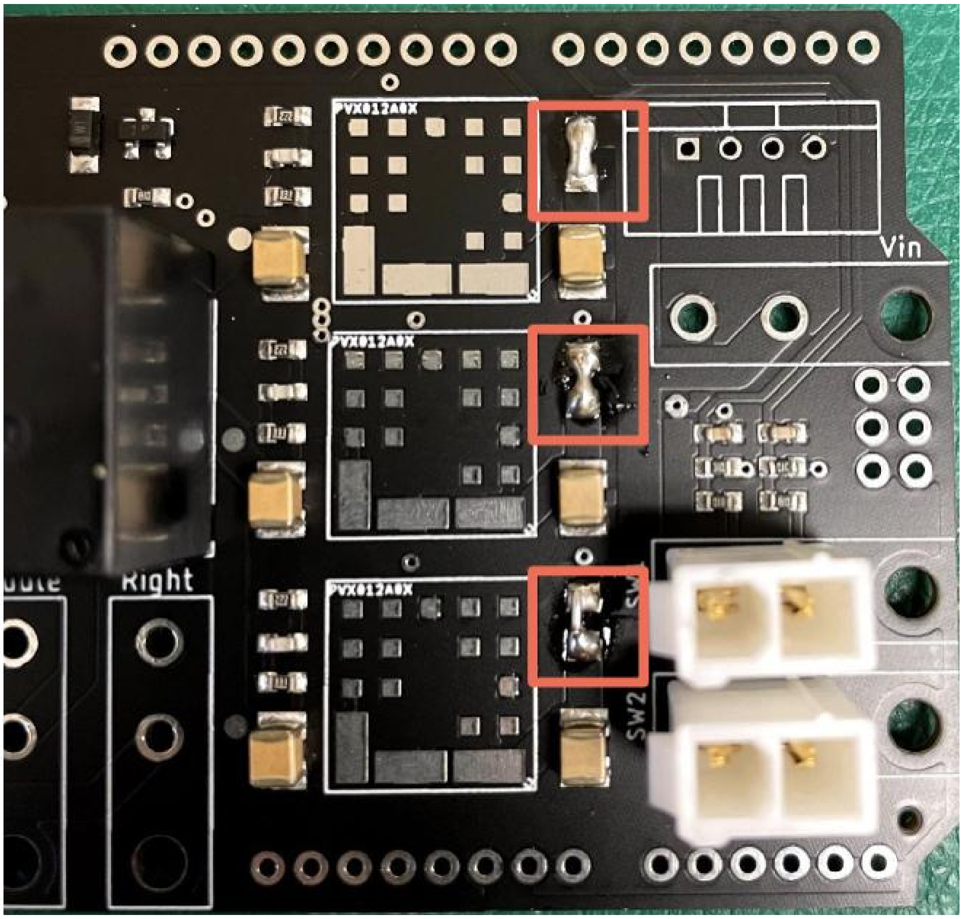

Figure SN24: Step #5 Solder three wires onto the pads above the capacitors

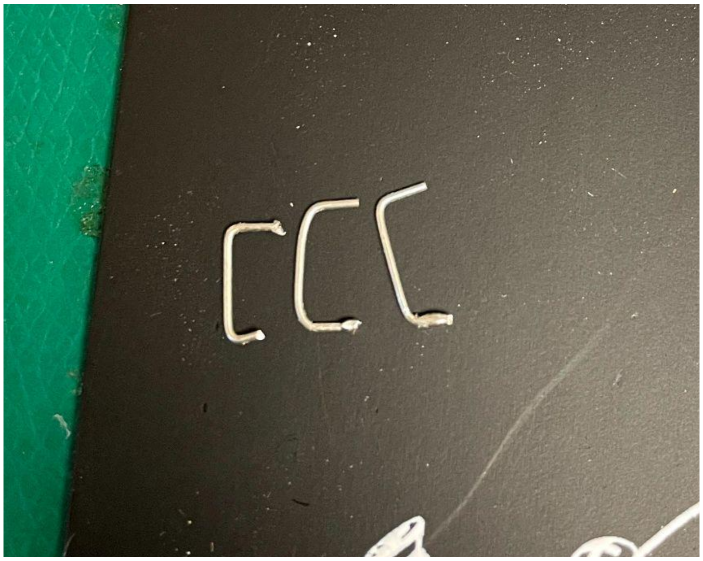

Figure SN25: Step #6 Cut another set of wire into 12 mm length, and bend them into a U-shape.

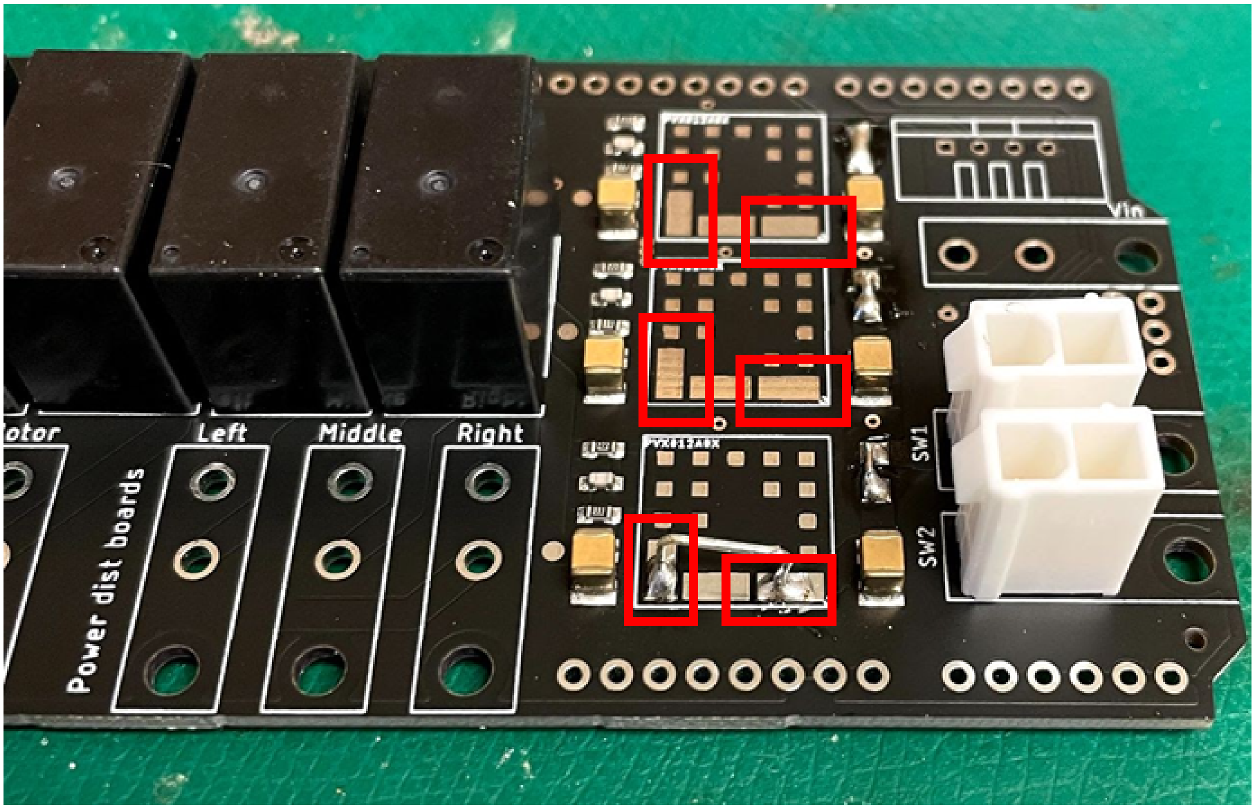

Figure SN26: Step #7 Solder a new set of wire between two big pads in the red boxes.

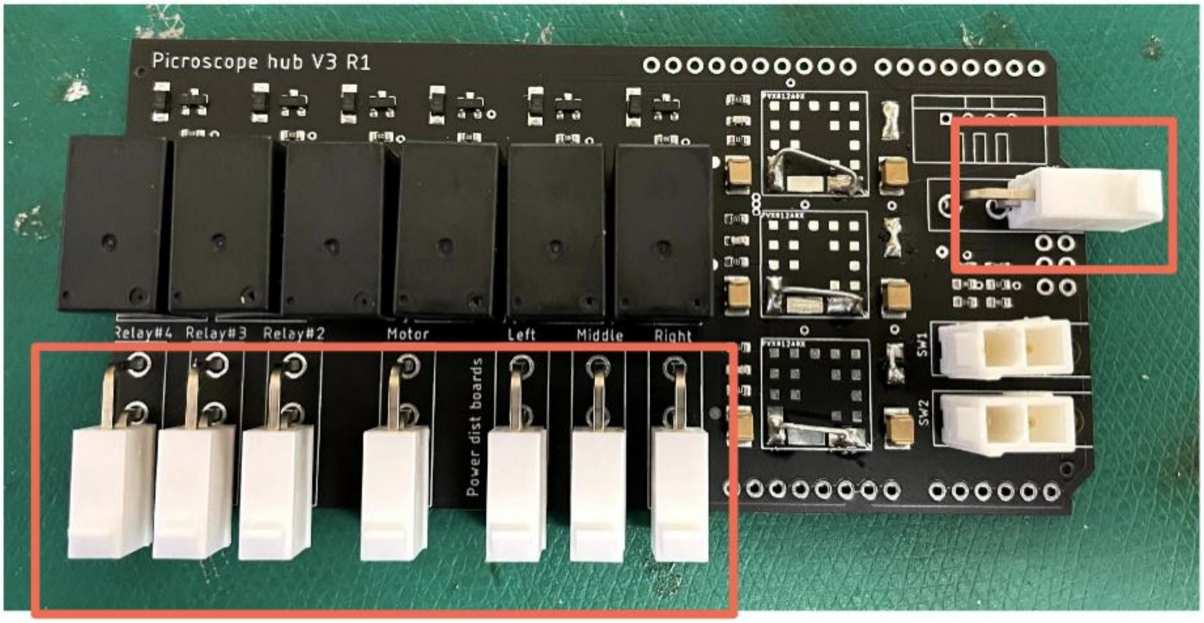

Figure SN27: Step #8 Solder the molex connector (Part number 0039301022) as seen in red.

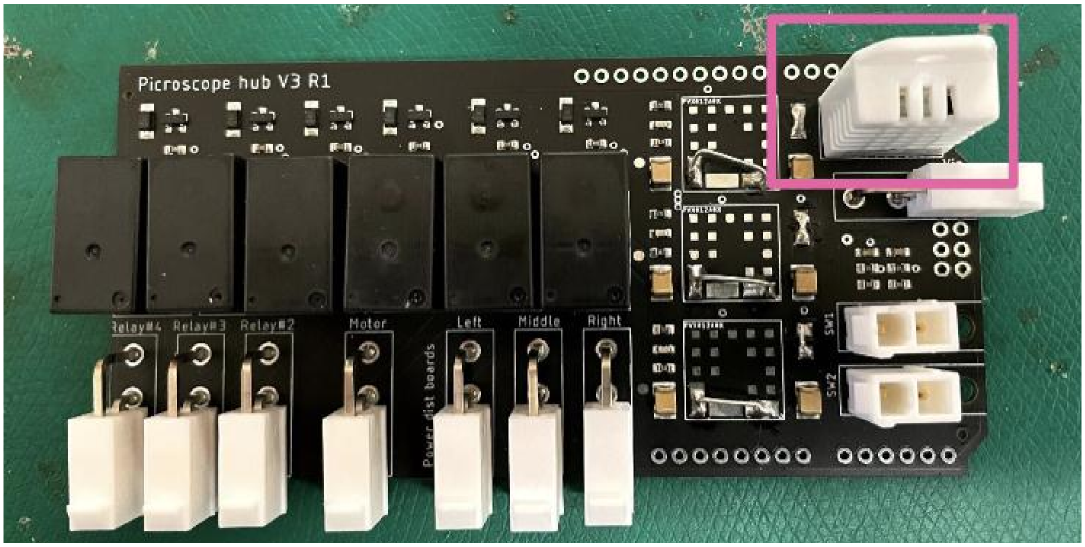

Figure SN28: Step #9 Solder the temperature sensor in pink (Part number 528-1504-ND)

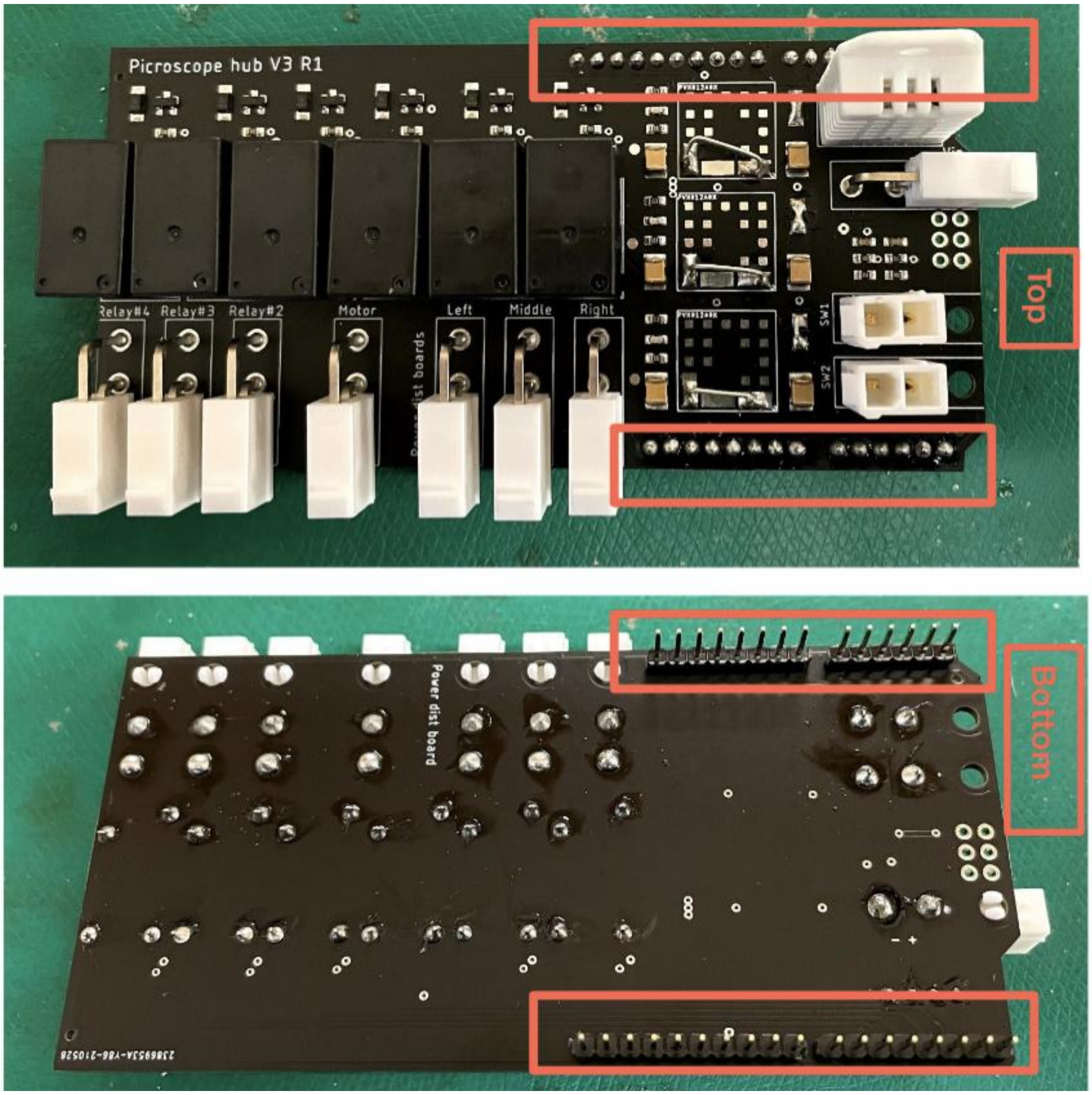

Figure SN29: Step #10 Solder the pin headers in red.

Wire assembly

Black wire: Part number 2153401122
Red wire: Part number 2153402122
Connector: Part number 0039039022

Note: black wire is close to connector clip

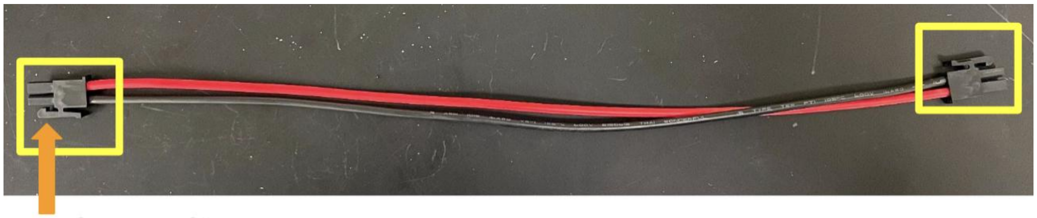

Figure SN30: Wire assembly
