# Supplementary material for: Picroscope: low-cost system for simultaneous longitudinal biological imaging
Source: Commun Biol. 2021 Nov 4;4:1261. doi: 10.1038/s42003-021-02779-7 (PMC8569150; doi:10.1038/s42003-021-02779-7)
Supplement: Supplementary file 2 — Description of Additional Supplementary Files [file 42003_2021_2779_MOESM2_ESM.pdf]

Description of Additional Supplementary Files

File name: Supplementary Video 1.
Description: Video Imaging of a Developing Zebrafish.

File name: Supplementary Data 1.
Description: Source data for charts in the main figures.
