# Supplementary material for: Picroscope: low-cost system for simultaneous longitudinal biological imaging
Source: Commun Biol. 2021 Nov 4;4:1261. doi: 10.1038/s42003-021-02779-7 (PMC8569150; doi:10.1038/s42003-021-02779-7)
Supplement: Supplementary file 5 — Reporting Summary [file 42003_2021_2779_MOESM5_ESM.pdf]

nature portfolio

Corresponding author(s): Victoria Ly, Mircea Teodorescu

Last updated by author(s): Sep 10, 2021

Reporting Summary

Nature Portfolio wishes to improve the reproducibility of the work that we publish. This form provides structure for consistency and transparency in reporting. For further information on Nature Portfolio policies, see our Editorial Policies and the Editorial Policy Checklist.

Statistics

For all statistical analyses, confirm that the following items are present in the figure legend, table legend, main text, or Methods section.

n/a	Confirmed	
☐	☒	The exact sample size (*n*) for each experimental group/condition, given as a discrete number and unit of measurement
☐	☒	A statement on whether measurements were taken from distinct samples or whether the same sample was measured repeatedly
☒	☐	The statistical test(s) used AND whether they are one- or two-sided<br>*Only common tests should be described solely by name; describe more complex techniques in the Methods section.*
☒	☐	A description of all covariates tested
☒	☐	A description of any assumptions or corrections, such as tests of normality and adjustment for multiple comparisons
☒	☐	A full description of the statistical parameters including central tendency (e.g. means) or other basic estimates (e.g. regression coefficient) AND variation (e.g. standard deviation) or associated estimates of uncertainty (e.g. confidence intervals)
☒	☐	For null hypothesis testing, the test statistic (e.g. *F*, *t*, *r*) with confidence intervals, effect sizes, degrees of freedom and *P* value noted<br>*Give P values as exact values whenever suitable.*
☒	☐	For Bayesian analysis, information on the choice of priors and Markov chain Monte Carlo settings
☒	☐	For hierarchical and complex designs, identification of the appropriate level for tests and full reporting of outcomes
☒	☐	Estimates of effect sizes (e.g. Cohen's *d*, Pearson's *r*), indicating how they were calculated

Our web collection on statistics for biologists contains articles on many of the points above.

Software and code

Policy information about availability of computer code

Data collection	All the code needed for data acquisition was custom made and it is available in the Github Repositoryhttps://github.com/braingeneers/picroscope-supplement/tree/main/Code
Data analysis	All the code needed for data analysis was custom made and it is available in the Github Repositoryhttps://github.com/braingeneers/picroscope-supplement/tree/main/Code

For manuscripts utilizing custom algorithms or software that are central to the research but not yet described in published literature, software must be made available to editors and reviewers. We strongly encourage code deposition in a community repository (e.g. GitHub). See the Nature Portfolio guidelines for submitting code & software for further information.

Data

Policy information about availability of data

All manuscripts must include a data availability statement. This statement should provide the following information, where applicable:

- Accession codes, unique identifiers, or web links for publicly available datasets
- A description of any restrictions on data availability
- For clinical datasets or third party data, please ensure that the statement adheres to our policy

All files needed for the design (CAD) of the Picroscope can be accessed in the Github Repositoryhttps://github.com/braingeneers/picroscope-supplement/tree/main/CADs. All files needed for the design of the circuit boards(PBC) can be accessed in the Github Repository https://github.com/braingeneers/picroscope-supplement/tree/main/PCB

Field-specific reporting

Please select the one below that is the best fit for your research. If you are not sure, read the appropriate sections before making your selection.

☒ Life sciences ☐ Behavioural & social sciences ☐ Ecological, evolutionary & environmental sciences

For a reference copy of the document with all sections, see nature.com/documents/nr-reporting-summary-flat.pdf

Life sciences study design

All studies must disclose on these points even when the disclosure is negative.

Sample size	All fertilized eggs of a frog mating were used. For planaria, zebrafish and stem-cell derived models, representative images were taken.
Data exclusions	For blastopore measurements we selected a subset of 27 eggs that had the blastopore within the focal planes of the scope.
Replication	N/A
Randomization	N/A
Blinding	N/A

Reporting for specific materials, systems and methods

We require information from authors about some types of materials, experimental systems and methods used in many studies. Here, indicate whether each material, system or method listed is relevant to your study. If you are not sure if a list item applies to your research, read the appropriate section before selecting a response.

Materials & experimental systems

n/a	Involved in the study	
☒	☐	Antibodies
☐	☒	Eukaryotic cell lines
☒	☐	Palaeontology and archaeology
☐	☒	Animals and other organisms
☒	☐	Human research participants
☒	☐	Clinical data
☒	☐	Dual use research of concern

Methods

n/a	Involved in the study	
☒	☐	ChIP-seq
☒	☐	Flow cytometry
☒	☐	MRI-based neuroimaging

Eukaryotic cell lines

Policy information about cell lines

Cell line source(s)	The H9 hESC line was obtained from WiCell.
Authentication	The cell lines used were authenticated
Mycoplasma contamination	The cells were routinely tested for mycoplasma and they tested negative.
Commonly misidentified lines (See ICLAC register)	N/A

Animals and other organisms

Policy information about studies involving animals; ARRIVE guidelines recommended for reporting animal research

Laboratory animals	Frog eggs were Xenopus tropicalis; Zebrafish eggs were Danio rerio; Planaria were Dugesia tigrina. We did not obtain sex information fro these animals.
Wild animals	N/A
Field-collected samples	N/A
Ethics oversight	Adult frogs were maintained according to approved UCSF IACUC protocols. Planaria worms and zebrafish eggs are deemed exempt from IACUC protocols.

Note that full information on the approval of the study protocol must also be provided in the manuscript.
